# Malaria and related outcomes in patients with intestinal helminths: a cross-sectional study

**DOI:** 10.1186/1471-2334-12-291

**Published:** 2012-11-09

**Authors:** Abraham Degarege, Mengistu Legesse, Girmay Medhin, Abebe Animut, Berhanu Erko

**Affiliations:** 1 Aklilu Lemma Institute of Pathobiology, Addis Ababa University, P.O. Box 1176, Addis Ababa, Ethiopia

**Keywords:** Non-severe malaria, Association, Intestinal helminths, *Plasmodium*, Co-infections, Ethiopia

## Abstract

**Background:**

The effects of helminth co-infection on malaria in humans remain uncertain. This study aimed to evaluate the nature of association of intestinal helminths with prevalence and clinical outcomes of *Plasmodium* infection.

**Methods:**

A cross-sectional study involving 1,065 malaria suspected febrile patients was conducted at Dore Bafeno Health Center, Southern Ethiopia, from December 2010 to February 2011. *Plasmodium* and intestinal helminth infections were diagnosed using Giemsa-stained blood films and Kato-Katz technique, respectively. Haemoglobin level was determined using a haemocue machine.

**Results:**

Among 1,065 malaria suspected febrile patients, 28.8% were positive for *Plasmodium* parasites (*P. falciparum* =13.0%, *P. vivax* =14.5%, *P. falciparum* and *P. vivax* =1.3%). Among 702 patients who provided stool samples, 53.8%, 31.6% and 19.4% were infected with intestinal helminths, *Plasmodium* alone and with both *Plasmodium* and intestinal helminths, respectively. The prevalence of infections with *Ascaris lumbricoides* (*A. lumbricoides*), *Trichuris trichiura* (*T. trichiura*), *Schistosoma mansoni* (*S. mansoni*) and hookworm (9.8%) were 35.9%, 15.8%, 11.7% and 9.8%, respectively. Out of the 222 (31.6%) *Plasmodium* infected cases, 9 (4.1%) had severe malaria. *P. falciparum* infection was more common in febrile patients infected with *A. lumbricoides* alone (21.3%), *T. trichiura* alone (23.1%) and *S. mansoni* alone (23.1%) compared to those without intestinal helminth infections (9.3%) (*p*<0.001 for all). Prevalence of non-severe malaria was significantly higher in individuals infected with intestinal helminths than in those who were not infected with intestinal helminths (adjusted OR=1.58, 95% CI=1.13-2.22). The chance of developing non-severe *P. falciparum* malaria were 2.6, 2.8 and 3.3 times higher in individuals infected with *A. lumbricoides* alone, *T. trichiura* alone and *S. mansoni* alone, respectively, compared to intestinal helminth-free individuals (*p*<0.05 for all). The odds ratio for being infected with non-severe *P. falciparum* increased with the number of intestinal helminth species (*p*<0.001). Mean *Plasmodium* density among intestinal helminth infected individuals was significantly increased with the number of intestinal helminths species (*p*=0.027). Individuals who were co-infected with different species of intestinal helminths and *Plasmodium* showed lower mean haemoglobin concentration than individuals who were infected only with *Plasmodium*.

**Conclusions:**

Infections with *A. lumbricoides, T. trichiura* and *S. mansoni* were positively associated with *P. falciparum* infection. However, further studies are required to investigate how these helminths could contribute to increased prevalence of *P. falciparum* infection.

## Background

Although *Plasmodium* and helminth co-infections are prevalent in tropical countries, the effect of their interactions remains unclear [1]. Some studies reported that helminth infected individuals are susceptible to *Plasmodium* infection [2-4], increased malaria gametocyte carriage [5], decreased heamoglobin concentration [6] and increased risk for clinical and severe malaria [7-9]. Cross-sectional studies conducted in Zaire and Zimbabwe showed a significant positive association between the prevalence of *P. falciparum* malaria and intestinal helminths infection [10,11]. Positive association was also observed between *Plasmodium* density and intensity of hookworm infection [12].

On the other hand, evidence from other studies showed that helminth infections may protect from malaria or related clinical outcomes by suppressing acute clinical manifestations [13,14], parasite density in human blood [15,16] or severe complications such as cerebral malaria, circulatory collapse, renal failure and jaundice [12,17-19].

Still other studies reported the absence of significant association between helminth infection and malaria [3,20]. These contradictory results could arise from the complex nature of the immune response to malaria parasites due to the variation in the *Plasmodia* stages and species or altered immune response because of helminth co-infections [1]. In addition, variations in study methodology, study design, case definition or malaria severity status, stage or intensity or species of helminths or *Plasmodium*, epidemiological setting of the study area and other confounding factors could contribute for the contradictions.

In Ethiopia, previous similar study was conducted in an area where infection with *P. falciparum* was less common (only 4.0%), and infection with *S. mansoni* was absent [12]. In addition, in that previous study, intestinal helminth infections and haemoglobin concentration were determined only for *Plasmodium* infected cases, and factors such as age, sex, nutrition and socioeconomic status were not considered in the analysis. Thus, the current study was conducted to assess the nature of association of intestinal helminths with prevalence and clinical outcomes of *Plasmodium* infection among malaria suspected febrile individuals attending Dore Bafeno Health Center, Southern Ethiopia.

## Methods

### Study area and participants

A cross-sectional study involving 1,065 febrile patients (age range= 1 to 82 years) was conducted at Dore Bafeno Health Center, Sidama Zone, southern Ethiopia, December 2010 to February 2011. The health center is located in Dore Bafeno District, about 283 km south of Addis Ababa, at about 1,708 m above sea level and 7° 5' N and 38° 29 E. It has a mean annual rainfall of 80.4 mm (it varies from 15 mm to 130 mm) and mean annual temperature of 23.2°C (it varies from 21.5°C to 25°C). The study participants were malaria suspected febrile patients who did not take anti-malarial drug within the last two weeks. Almost all the study participants belonged to the Sidama ethnic group, residing in rural area with similar life style and earning their living as mixed-farming. Malaria is unstable in the area and occurs mainly from October to December following the heavy rainy season and from April to May following the light rain season (health facility reports).

### Socio-demographic and clinical data

Clinical data including auxiliary body temperature, blood pressure, coma status, breathing status and prostration were collected by physicians. Clinical data together with laboratory results were used to group malaria cases as severe and non-severe [21]. Socio-demographic data including age, sex, height (to the nearest 0.1 cm) and weight (to the nearest 0.1 kg) were also recorded. Nutritional indicators (i.e. z-scores) were calculated using anthro [22] for children age < 5 and using anthro-Plus [23] for children age between 5 and 19 years. Each child was classified as being under-nourished (if z-score is less than −2) or well-nourished (if z-score is at least −2) [24]. For adults older than 19 years, the standard categories of body mass index (BMI<18.5=undernourished; BMI ≥ 18.5 well-nourished) were used to determine the nutritional status [25].

### Malaria microscopy and haemoglobin determination

Approximately, 20μl finger prick blood was collected from each febrile patient using a plastic capillary tube and 10 μl of the blood was used to prepare thick and thin blood films on a single slide for the diagnoses and count of malaria parasitaemia [26]. The slides were re-examined for quality assurance. The remaining blood sample was used to determine haemoglobin level using HemoCue HB 201, Anghelom, Sweden.

### Collection and examination of stool samples

Fresh stool specimens were collected from each study participant who also gave blood sample for malaria diagnosis. The stool specimens were prepared for microscopic examination using double Kato slides [27]. Quantitative examination for hookworms was made within 45 minutes of stool collection, whereas *T. trichiura*, *S. mansoni* and *A. lumbricoides* infections were quantified at the Aklilu Lemma Institute of Pathobiology (ALIPB), Addis Ababa University. Average egg counts of the two slides was multiplied by 24 to obtain egg counts per gram of stool for each of the study participants and classes of intensity was determined for different helminth species as per the WHO recommendation [28].

### Ethical consideration

This study was conducted after obtaining ethical clearance from the Institutional Review Board (IRB) of Aklilu Lemma Institute of Pathobiology, Addis Ababa University. As the majority of the population in the study area was illiterate the IRB endorsed oral consenting of the study participants. Permission to conduct the study was also obtained from Awassa Zuria District Health Office and Dore Bafeno Health Center. Detailed explanations about the aims, procedures, potential risks and benefit of the study were given to physicians of the health center. Individual oral consent was obtained in local language by the physicians of the health center during clinical examination and only voluntarily consented individuals were included in the study. For children younger than 18 years, assent was obtained from parents or guardians. Treatment was made based on the national protocol for treating malaria and helminth infections. Individuals who were found positive for *P. falciparum* and *P. vivax* infection were treated with coarteum and chloroquine, respectively. Individuals who were found positive for the soil-transmitted helminths (STHs) were treated with 400mg of albendazole while praziquantel was used to treat those who were positive for *S. mansoni*, *Taenia saginata* (*T. saginata*), and *Hymenolepis nana* (*H. nana*). The study participants were treated free of charge. Since the study participants were selected from patients coming to the health center seeking medical treatment for malaria, they were not compensated for extra incentive to replace their transport cost or time lost from work was not given.

### Data analysis

Data was computerized using Epi-data version 3.1 software (The EpiData Association, Odense, Denmark) and analyzed using STATA version 11 (Stata Corporation, College Station, Texas, USA). Pearson χ^2^-test and one-way ANOVA were used to compare malaria prevalence and mean parasitaemia or egg intensity values, respectively across categories of categorical variables. Univariable and multivariable logistic regression analyses were used to test for an association between intestinal helminths infection and prevalence of non-severe malaria or malaria related anaemia. Multiple linear regression analyses was used to evaluate differences in the means of *Plasmodium* density and haemoglobin level between *Plasmodium*-helminth co infected and those who were infected with *Plasmodium* alone. The analysis was adjusted for age, sex and nutritional status [29,30], while the association between helminth and *Plasmodium* infections, or related outcomes was evaluated. Since the study population was homogenous in their socio-economic status differences in terms of socioeconomic status were not considered during the analysis. The distribution of parasite density was skewed and square root transformation was used to make it symmetrical before using linear regression to evaluate the effect of pre-specified risk factors on this outcome. Results were considered significant whenever p*-*value was less than 5%.

## Results

### Prevalence of malaria

Among 1,065 malaria suspected febrile patients (Mean age=18.6 years), 306 (28.8%) were positive for *Plasmodium* parasites (Table 1). *Plasmodium* infection was higher among febrile patients in the 5–15 years age group and in the under 5 children compared to individuals older than 15 years (*p*<0.001). *P. falciparum* infection alone or mixed infection with *P. vivax* was also higher in the 5–15 years old children compared to in individuals older than 15 years (*p*<0.01). Infection with *P. vivax* alone was higher (*p*<0.001) in the under five children and in the 5–15 years age group compared to those individuals older than 15 years. There was no significant difference in the percentage of *P. falciparum* and/or *P. vivax* infection between males and females.

**Table 1:** Prevalence of malaria among malaria suspected febrile patients who attended Dore Bafeno Health Center, southern Ethiopia, December 2010

**Variables**	**Number examined**	***P.falciparum*positive n (%)**	***P. vivax*positive n (%)**	***P. falciparum*&*P. vivax*positive n (%)**	**Total malaria positive n (%)**
Age					
<5	255	28 (11.0)	57 (22.4)	3 (1.2)	88 (34.5)
5–15	240	56 (23.3)	39 (16.3)	7 (2.9)	102 (42.5)
>15	570	54 (9.5)	58 (10.2)	4 (0.7)	116 (20.4)
χ^2^ (*p*)		36.12 (<0.001)	26.35 (<0.001)	9.69 (0.008)	45.91 (<0.001)
Sex					
Female	522	64 (12.3)	68 (13.0)	8 (1.5)	140 (26.8)
Male	543	74 (13.6)	86 (15.8)	6 (1.1)	166 (30.6)
χ^2^ (*p*)		0.73 (0.393)	1.95 (0.162)	0.26 (0.613)	1.83 (0.176)

### Prevalence of helminths infection

Among 1,065 febrile patients examined for malaria, 702 were volunteered to provide stool samples for the diagnosis of infection with intestinal helminths. There was no significant difference in baseline demographics between those who provided stool sample and those who did not (data not shown). Out of 702 patients examined, 53.8% were positive for at least one intestinal helminth species (Table 2). The diagnosed helminth species were *A. lumbricoides* (35.9%), *T. trichiura* (15.8%), *S. mansoni* (11.7%), hookworm (9.8%), *T. saginata* (3.9%), *H. nana* (1.4%) and *Enterobius vermicularis* (*E. vermicularis*) (0.9%). Of the 378 intestinal helminth infected individuals, 35.8%, 13.3%, 4.3% and 0.6% had single, double, triple and quadruple infections, respectively. Intestinal helminth infection was most prevalent among individuals of age 5–15 years (63.2%). *A. lumbricoides* and *T. trichiura* infections were higher among individuals in 5–15 years age group than among individuals of age >15 years.

**Table 2:** Intestinal helminth infection among malaria suspected febrile patients Dore Bafeno Health Center, southern Ethiopia, December 2010

**Variable**	**Number examined**	***A.lumbricoides*n (%)**	**Hookworm n (%)**	***T. trichiura*n (%)**	***S. mansoni*n (%)**	Others* n (%)	Any intestinal helminth infection** n (%)
Age							
<5	88	24 (27.3)	2 (2.3)	12 (13.6)	3 (3.4)	6 (6.8)	35 (39.3)
5–15	182	86 (47.3)	10 (5.5)	36 (19.8)	24 (13.2)	9 (5.0)	115 (63.2)
>15	432	142 (32.9)	57 (13.2)	63 (14.6)	55 (12.7)	15 (3.5)	228 (52.8)
χ^2^ (*p*)		14.76 (0.001)	15.05 (0.001)	2.96 (0.228)	6.70 (0.035)	5.96 (0.202)	14.13 (0.001)
Sex							
Female	341	135 (39.6)	28 (8.2)	65 (19.1)	33 (9.7)	19 (5.6)	190 (55.72)
Male	361	117 (32.4)	41(11.4)	46 (12.7)	49 (13.6)	11 (3.0)	188 (51.93)
χ^2^ (*p*)		3.93 (0.047)	1.96 (0.162)	5.26 (0.022)	2.58 (0.108)	3.06 (0.217)	1.02 (0.314)

Others* = infection with either of *H. nana*, *T. Saginata*, *E. vermucularis*.

Any intestinal helminth infection**= infection with at least one intestinal helminth species.

Mean intensity of infections were 2345 egg per gram {epg} (range; 24–16128) for *A. lumbricoides*, 372 epg (range; 24–12816) for *S. mansoni*, 107 epg (range; 24–1200) for hookworm and 101 epg (range; 24–1296) for *T. trichiura*. Intensity of *A. lumbricoides* infection showed age related pattern with the highest (F=3.36, *p*=0.036) mean intensity being observed among individuals of age group 5–15 years (mean epg=3113.7) followed by individuals of age group <5 years (mean epg=2410.6). Mean intensity of hookworm infection was higher (F=15.69, *p*<0.001) among individuals of age <5 years (mean epg=648) followed by those individuals whose age ranges from 5–15 years (mean epg=69.6).

### Malaria and helminth co-infection

Out of 702 patients, 242 (34.5%) were infected with intestinal helminths alone, 86(12.3%) with *Plasmodium* alone, and 136 (19.4%) with both *Plasmodium* and intestinal helminths (Table 3). Intestinal helminthic infection was significantly associated with increased odds of malarial infection (i.e. *P. falciparum* and/or *P. vivax* infection) (unadjusted OR =1.56, 95% CI=1.13-2.16, *p*=0.007). Prevalence of *P. falciparum* infection was significantly higher in individuals infected with any intestinal helminth (s), *A. lumbricoides* alone, *T. trichiura* alone, *S. mansoni* alone compared to those who were not infected with intestinal helminths (9.3%) (*p*<0.001 for all) (Table 3).

**Table 3:** Prevalence of malaria and intestinal helminth co-infections among malaria suspected febrile patients who attended Dore Bafeno Health Center, southern Ethiopia, December 2010

**Helminth infection**	**Number of cases**	***P. falciparum*positive n (%)**	***P. vivax*positive n (%)**	***P. falciparum*and*P. vivax*n (%)**	**Total malaria positive n (%)**
Only *A. lumbricoides* (*Al*)	141	30 (21.3)	20 (14.2)	2 (1.4)	52 (36.9)
Only hookworm (*Hw*)	25	1(4.0)	6 (24.0)	0	7 (28.0)
Only *T. trichiura* (*Tt*)	39	9 (23.1)	6 (15.4)	0	15 (38.5)
Only *S. mansoni* (*Sm*)	39	9 (23.1)	3 (7.7)	0	15 (38.5)
*Al* and *Tt*	42	9 (31.0)	11 (35.5)	2 (4.5)	22 (52.4)
*Al* and *Sm*	18	4 (22.2)	1 (5.6)	0	5 (27.8)
*Al*, *Tt* and *Sm*	5	1 (20.0)	1 (20.0)	0	2 (40.0)
*Al*, *Tt*, *Sm* and *Hw*	22	7 (31.8)	1 (4.5)	0	8 (36.4)
Any intestinal helminth	378	80 (21.2)	51(13.5)	5 (1.3)	136 (36.0)
Without intestinal helminths	324	30 (9.3)	50 (15.4)	6 (1.9)	86 (26.5)

Out of 306 *Plasmodium* infected patients, 14 showed one or more sign and symptoms of severe malaria (i.e. prostration in 10 patients, breathing difficulty syndrome in 8 patients, cerebral malaria in 7 patients and severe anaemia in 3 patients). Nine severe malaria cases were volunteered to provide stool samples for the diagnosis of helminth infection and 7 of them were co-infected with intestinal helminths like *A. lumbricoides*, *T. trichiura* and hook worm.

Of the 292 non-severe malaria cases, 213 patients were examined for intestinal helminth infection and 61.0% were positive. The odds of non-severe malaria infection was higher in individuals infected with intestinal helminth compared to intestinal helminth-free individuals {adjusted Odds Ratio (AOR) =1.57, 95% CI= 1.12, 2.21}. Similarly, the odds of non-severe *P. falciparum* mono-infection was higher among individuals infected with intestinal helminth than non-infected with intestinal helminth (AOR=2.89, 95% CI=1.77, 4.72). The ORs changed in parallel with the number of intestinal helminth species infection (p<0.001). Individuals infected with *A. lumbricoides* alone (AOR=2.6), *T. trichiura* alone (AOR=2.8), *S. mansoni* alone (AOR=3.3), any intestinal helminth (AOR=2.9), *T. trichiura* and *A. lumbricoides* (AOR=3.1), and *T. trichiura*, *A. lumbricoides*, *S. mansoni* and hookworm (AOR=4.0) had increased odds of non-severe *P. falciparum* mono-infection compared to those who were not infected with any intestinal helminths (*p*<0.05 for all) (Table 4). However, the proportions of *P. vivax* mono-infected individuals were not significantly different when compared between those who had single or multiple infections with different intestinal helminths and intestinal helminth free individuals (data not shown). Similarly, infection with hookworm alone was not significantly associated with any of non-severe *P. falciparum* and/or *P. vivax* infection.

**Table 4:** **Association of intestinal helminth infection and non-severe*P. falciparum*malaria among malaria suspected febrile patients who attended Dore Bafeno Health Center, southern Ethiopia, December 2010**

**Outcome**	**Main exposure**	**Model**	**OR**	**95% CI**	**p-value**
*P. falciparum*	Only *A. lumbricoides* (*Al*)	Crude	2.58	1.44, 4.61	0.001
		Adjusted*	2.55	1.40, 4.63	0.002
	Only hookworm (*Hw*)	Crude	0.47	0.06, 3.67	0.473
		Adjusted*	0.54	0.07, 4.35	0.565
	Only *T. trichiura* (*Tt*)	Crude	2.48	0.98, 6.27	0.055
		Adjusted*	2.77	1.15, 6.65	0.023
	Only *S. mansoni* (*Sm*)	Crude	2.83	1.21, 6.63	0.016
		Adjusted*	3.25	1.32, 7.97	0.010
	*Al* and *Tt*	Crude	3.57	1.49, 8.55	0.004
		Adjusted*	3.12	1.27, 7.61	0.013
	*Al* and *Sm*	Crude	2.44	0.75, 7.96	0.139
		Adjusted*	2.65	0.76, 9.22	0.125
	*Al*, *Tt* and *Sm*	Crude	2.64	0.27, 26.23	0.406
		Adjusted*	2.34	0.23, 23.88	0.473
	*Al*, *Tt*, *Sm* and *Hw*	Crude	3.96	1.48, 10.60	0.006
		Adjusted*	4.12	1.49, 11.31	0.006
	Any intestinal helminth	Crude	2.74	1.71, 4.41	0.000
		Adjusted*	2.89	1.77, 4.72	0.000

NB 1. Individuals free from any intestinal helminth infection were used as reference categories while calculating the OR.

2. Adjusted*=the model is adjusted for the effects of age, gender and nutritional status.

3. Any intestinal helminth *=* individuals infected with at least 1 intestinal helminth species.

Data regarding the effect of intestinal helminths co infection on malaria-related outcomes are summarized in Table 5. After adjusting for age, sex and nutritional status mean *Plasmodium* density was significantly higher in individuals co-infected with four different intestinal helminth species (*T. trichiura*, *A. lumbricoides*, *S. mansoni* and hookworm) compared to those who were free from intestinal helminth infection (β = 10829.56, *p*<0.05). Similarly, there was an increasing trend in mean *Plasmodium* parasitaemia among individuals who were infected with *T. trichiura* alone, *S. mansoni* alone, *A. lumbricoides* alone and any two or three of these species compared to individuals who were not infected with any intestinal helminth species. The mean *Plasmodium* density among intestinal helminth infected individuals increased significantly as the number of intestinal helminth species increased (F=2.82, *P*=0.027). However, patients infected with hookworm alone showed slightly lower mean *Plasmodium* parasitaemia compared to individuals who had no intestinal helminth infection.

**Table 5:** Effect of intestinal helminth infection on malaria related outcomes among microscopic confirmed malaria cases who attended Dore Bafeno Health Center, southern Ethiopia, December 2010 to February 2011

		**Outcomes**	
**Helminth infection**	*Plasmodium*density*	**Haemoglobin**	**Anaemia**
	β *** [95% CI]	β *** [95% CI]	AOR** [95% CI]
*A. lumbricoides* (*Al*)	2.34 [−24.68, 20.00]	−0.52 [−1.37, 0.32]	1.53 [0.72, 3.22]
Hookworm (*Hw*)	−11.75 [−83.76, 60.26]	−0.65 [−2.64 , 1.34]	1.69 [0.33, 8.81]
*S. mansoni* (*Sm*)	3.65 [−35.09, 42.41]	0.12 [−1.45, 1.68]	1.11 [0.30, 4.17]
*T. trichiura* (*Tt*)	19.11 [−13.31, 51.55]	−0.65 [−2.64 , 1.34]	1.69 [0.33, 8.81]
*Al* and *Tt*	15.16 [−21.34, 51.67]	-.63 [−1.77, 0.50]	2.31 [0.80, 6.67]
*Al* and *Sm*	22.19 [−55.82, 100.19]	-.08 [−2.68, 2.52]	1.27 [0.17, 9.47]
*Al*, *Tt* and *Sm*	64.65 [−10.53, 139.85]	−2.35 [−6.35, 1.63]	2.83 [0.98, 15.24]
*Al*, *Tt*, *Sm* and *Hw*	52.05 [5.08, 99.03]	−1.62 [−3.61, 0.37]	8.64 [1.18, 63.47]
Any intestinal helminth	14.51 [−3.06, 32.07]	−0.34 [−0.99, 0.30]	1.54 [0.81, 2.90]

NB 1.helmith free but *Plasmodium* infected individuals are used as reference for calculating AOR-values and regression coefficients.

2. *Plasmodium* density*= *Plasmodium* density is on square root scale.

3. AOR** odds ratio from multivariate logistic regression models adjusted for effects of age, gender and nutritional status.

4. β *** regression coefficient from multiple linear regression models adjusted for effects of age, gender and nutrition.

Individuals co-infected with *A. lumbricoides,* hookworm*, S. mansoni*, *T. trichiura* alone or in combination and *Plasmodium* showed lower mean haemoglobin levels compared to malaria cases without intestinal helminths infections. However, the mean differences were not significant in the multivariable model. Prevalence of anaemia was higher among individuals co-infected with both intestinal helminths and *Plasmodium* compared to those with *Plasmodium* infection alone. Individuals co-infected with *Plasmodium* and four different intestinal helminths were almost 9 times more likely to be anaemic compared to those with *Plasmodium* infection alone (AOR=8.64, 95% CI=1.18, 63.47).

There was not significant correlation between the mean *Plasmodium* density and intensity of intestinal helminth infections (spearman correlation coefficient= 0.12, -0.19, 0.04, -0.08 for *A. lumbricoides,* hookworm, *T. trichiura, S. mansoni*, respectively). In *S. mansoni* and *T. trichiura* infected patients, the mean parasitaemia increased as the class of intensity of the helminths increased; nonetheless the regression coefficients remain non-significant after adjusting for the effects of age, sex, nutrition, and concurrent infection by other species of helminth. Conversely, the mean parasitaemia of individuals with moderate intensity of *A. lumbricoides* infection was relatively low compared to individuals co infected with light intensity of *A. lumbricoides* and *Plasmodium*. For hookworm infection, all the infected cases were of light intensity class (data not shown).

The reduction in mean haemoglobin level among individuals co-infected with intestinal helminths and *Plasmodium* was negatively correlated with intensity of intestinal helminths. However, the coefficients for the association were low. Similarly, although the mean differences were not significant, haemoglobin levels decreased more in *Plasmodium* infected individuals co-infected with heavy intense *S. mansoni* or moderate intense *T. trichiura* infections compared to individuals with light intensity of infections with the respective intestinal helminths (data not shown).

## Discussion

In this study, the likelihood of being infected with non-severe *P. falciparum* malaria was significantly higher in individuals infected with intestinal helminths, particularly in those with *A. lumbricoides* alone, *T. trichiura* alone or *S. mansoni* alone compared to individuals without intestinal helminths. Similarly, prevalence of non-severe malaria (*P. falciparum* and/or *P. vivax*) was significantly higher in individuals infected with intestinal helminth or *A. lumbricoides* alone compared to individuals without intestinal helminths. These findings are similar with previous finding in children [9,11] and adults [10]. Increased incidence of *P. falciparum* malaria was also previously reported in helminth infected individuals [3,7,8].

In concordance with previous reports [6,31], a relatively lower mean heamoglobin level was observed among *Plasmodium* and intestinal helminth co-infected patients compared to those with only *Plasmodium* infection. There was a non-significant decreasing trend in mean haemoglobin levels as the number of intestinal helminths species per an individual increased. Similarly, the association of anaemia was slightly higher in individuals co-infected with *Plasmodium* and intestinal helminth compared to malaria cases without having intestinal helminth infections. Intestinal helminths and malaria reduce haemoglobin level differently [32,33], implying that their effect will be additive in causing anaemia when they co-exist in a host.

Mean *Plasmodium* density was slightly higher among intestinal helminth infected individuals compared with intestinal helminth free individuals. These results together with the high odds of anaemia (or decreased mean haemoglobin levels) and non-severe *P. falciparum* and/or *P. vivax* infection give the impression of a positive association between intestinal helminth infection and non-severe malaria. This association between prevalence or outcomes of malaria and intestinal helminth infections seemed to increase with an increase in the number of intestinal helminths species. This is in agreement with previous reports from Ethiopia [12] and Thailand [3] where the risk of high *Plasmodium* density and *P. falciparum* infection, respectively increased with the number of intestinal helminth species.

The present findings appear to substantiate the hypothesis that clinical malaria can be aggravated during concomitant infection of intestinal helminths and *Plasmodium*[32]. Worms are known to induce regulatory T cells leading to the production of cytokines that counteracts Th1 response [34]. Helminth also modulates the function of denderitic cells, consequently altering the immune response to malaria antigen [1]. Additionally, helminth ligand repeatedly stimulate toll like receptors, thereby down regulating responsiveness to *Plasmodium* during concomitant infection [35]. Helminth infection could also make the skin less retort to mosquito bites [36] promoting the success of sporozoite to pass through it and increasing the chance of blood stage infection.

However, *Plasmodium* density was less associated with intensity of helminth infections and results regarding mean differences of *Plasmodium* density between intestinal helminth free and those infected with different intestinal helminth species were contradictory. Compared to intestinal helminth free malaria cases, the mean *Plasmodium* density of individuals infected with *A. lumbricoides* alone, *S. mansoni* alone or *T. trichiura* alone was slightly higher, but lower in the case of single hookworm infection. These controversial results on mean *Plasmodium* changes could have arisen due to the dominancy of light intense helminth infection among cases or complex nature of helminth and *Plasmodium* interaction when co-exist in a host.

Individuals with hookworm infection alone were neither protected nor associated with increased non-severe *P. falciparum* and/or *P. vivax* infection. This is in agreement with previous reports from Uganda [20] and Zaire [37], but in conflict with those from Zimbabwe [11]. The low infection intensity of hookworm (all cases light infection) in the present study might have contributed to the present finding. Had some of the cases been with moderate and/or heavy intensity classes the nature of the association could have been better explored. In addition, the present study revealed the absence of significant association between *P. vivax* mono-infection and infection with different intestinal helminth species which agrees with the observation in Thailand [18]. This could be due to the difference in pre-erythrocyte immunity for the two *Plasmodium* species [38].

Previous studies documented negative [3,17-19] and positive [8] association between geohelminth infection and malaria severity. However, the number of severe malaria cases in the current study was very low, limiting assessment about the nature of association between intestinal helminth infection and complicated malaria.

Variation in inferences about the nature of malaria and helminth interaction among reports could be due to differences in the nature of the study participants (children, pregnant, adults), study design, methodology and the way data was analyzed and interpreted. In most previous studies, data analysis did not separate the effect of different helminth or *Plasmodium* species. In addition, the effect of confounding factors such as age, sex, nutrition status, socioeconomic factors and other infections (e.g., HIV) were not usually considered in previous studies. It is well known that, level of endemicity (both helminths and malaria), local climate, traditional and modern health care practices as well as community health seeking behaviors vary from country to country and even from district to district. These variations could also bring substantial differences in helminth-malaria interactions and hence a result from one country/district might not be necessarily generalized or interpreted in another country/district where the above factors are significantly different.

Participants of the current study were more homogenous in that they were largely (>90% of the cases) similar in their life style, education level (illiterate), income level (average) and means of making a living (agriculture), place of residence (rural) and ethnicity (Sidama). However, slight differences in socioeconomic status might have existed among the cases which could partly affect the current data. In addition, cross-sectional nature of the study design has been a limitation to make conclusion about the risk or incidence of malaria due to previous status of helminth infection.

## Conclusions

In the current study, infections with *A. lumbricoides, T. trichiura* and *S. mansoni* were positively associated with *P. falciparum* infection. However, further studies are required to investigate how these helminths could contribute to increased prevalence of *P. falciparum* infection.

## Competing interests

The authors declare they have no competing interests.

## Authors’ contributions

AD: conceived the project idea and designed the study, collected, analyzed and interpreted the data. GM: involved in data analysis. AD, GM, ML, AA and BE involved in the drafting of the manuscript for important intellectual content. All authors read the draft manuscript and approved the final copy for submission.

## Pre-publication history

The pre-publication history for this paper can be accessed here:

http://www.biomedcentral.com/1471-2334/12/291/prepub
